# Persistent inflammation and T cell exhaustion in severe sepsis in the elderly

**DOI:** 10.1186/cc13941

**Published:** 2014-06-24

**Authors:** Shigeaki Inoue, Kodai Suzuki, Yukako Komori, Yukiko Morishita, Kyoko Suzuki-Utsunomiya, Katsuto Hozumi, Sadaki Inokuchi, Takehito Sato

**Affiliations:** 1 Institute of Innovative Science and Technology, Tokai University School of Medicine, Shimokasuya 143, Isehara, Kanagawa 259-1193, Japan; 2 Department of Emergency and Critical Care Medicine, Tokai University School of Medicine, Shimokasuya 143, Isehara, Kanagawa 259-1193, Japan; 3 Department of Emergency and Disaster Medicine, Graduate School of Medicine, Gifu University, 1-1 Yanagido, Gifu City, Gifu 501-1193, Japan; 4 Department of Immunology, Tokai University School of Medicine, Shimokasuya 143, Isehara, Kanagawa 259-1193, Japan

## Abstract

**Introduction:**

Sepsis is known as a complex immunological response with hyperinflammation in the acute phase followed by immunosuppression. Although aging is crucial in sepsis, the impact of aging on inflammation and immunosuppression is still unclear. The purpose of this study was to investigate the relationship between inflammation and immunosuppression in aged patients and mice after sepsis.

**Methods:**

Fifty-five patients with severe sepsis and 30 healthy donors were prospectively enrolled, and 90-day survival was compared between elderly (≥65 years) and adult (18–64 years) septic patients with serial measurement of serum interleukin (IL)-6. Within 24 h after diagnosis of severe sepsis, peripheral blood mononuclear cells were stimulated *ex vivo* to measure expression of the activation maker CD25 in T cells, IL-2 levels in the supernatant, and proliferation. In the mouse study, young (6–8 weeks) and aged (20–22 months) C57/B6 mice were subjected to cecal ligation and puncture (CLP), and survival was compared after 7 days with serial measurement of serum IL-6. Expression of the negative co-stimulatory molecules, CD25, and IL-2 in CD4+ T cells was measured.

**Results:**

The survival rate in elderly sepsis patients and aged septic mice was significantly lower than that in adult patients and young septic mice (60% vs. 93% in septic patients, 0% vs. 63% in septic mice, *P* < 0.05). Serum IL-6 levels in elderly sepsis patients and aged septic mice were persistently higher than those in adult patients and young septic mice. Expression of negative co-stimulatory molecules in CD4+ T cells in the spleen, lymph nodes, and peripheral blood was significantly higher in aged mice than in young mice (*P* < 0.01). *Ex vivo* stimulation decreased CD25 expression, IL-2 production, and proliferation to a greater extent in CD4+ T cells from elderly patients and aged septic mice than in those from adult patients and young septic mice. Elderly patients demonstrated increased detection of gram-negative bacteria at days 14–16 and 28–32 after sepsis (*P* < 0.05).

**Conclusions:**

Persistent inflammation and T cell exhaustion may be associated with decreased survival in elderly patients and mice after sepsis.

## Introduction

Sepsis, a systemic inflammatory response to infection, annually kills more than 210,000 people in the United States [1] and is one of the most challenging clinical problems worldwide, constituting the leading cause of death in non-coronary intensive care units [2]. Sepsis initiates a complex immunologic response that varies over time, with alternating predominance of both pro-inflammatory and anti-inflammatory mechanisms [3-6]. After a short pro-inflammatory phase, septic patients are thought to enter a stage of protracted immunosuppression [6].

Inflammation is characterized by excessive production of pro-inflammatory mediators [7], and immunosuppression is characterized by disturbed phagocytosis, antigen presentation by monocytes with decreased expression of HLA-DR and dysfunction, and apoptosis of lymphocytes, finally leading to shutdown of innate and adaptive immunity [8]. This phenomenon has recently been identified as an important cause of mortality during late-stage sepsis [6,9]. Recently, a genome-wide expression analysis of severely injured patients revealed that hyperinflammation and immunosuppression occur simultaneously [10], which introduced a new conceptual framework of persistent inflammation, immunosuppression, and catabolism syndrome (PICS) [11].

Although aging plays a critical role in the incidence and outcome of sepsis, the impact of aging on the coincidental inflammation and immunosuppression is still unclear. Nearly 60% of sepsis cases occur in patients aged over 65 years, who only comprise 12% of the population [12,13]. The average age of patients with sepsis is increasing, and age is a known independent predictor of mortality in sepsis patients [13,14]. We previously reported that elderly patients have reduced numbers of immunocompetent T cells and persistent lymphopenia with delayed death after sepsis [15]. However, we did not clarify the relationship between inflammation and immunosuppression in the elderly after sepsis and did not measure serum levels or *ex vivo* production of pro-and anti-inflammatory cytokines. Therefore, the purpose of this study was to further investigate the relationship between inflammation and immunosuppression in aged patients and mice after sepsis.

## Materials and methods

### Patient study

This study was approved by the ethics board of Tokai University Hospital in Japan. Patients with severe sepsis or septic shock and healthy donors (HDs) were prospectively included. Patients who had severe sepsis and had been admitted to either the emergency department or ICU were included after written informed consent was obtained from the patients or their next of kin. In this study, we used previously reported definitions of sepsis, severe sepsis, and septic shock [15]. Exclusion criteria included a lack of informed consent, age less than 18 years, pre-existing cancer, hematological or immunological disease, uncontrolled diabetes, pre-treatment with immunosuppressive agents, and chronic viral infections (human immunodeficiency virus or hepatitis B or C). For comparison, the donors and patients were divided into two groups based on age: adult (18 to 64 years) and elderly (≥65 years).

#### Antibodies

The following human antibodies (Bio-Legend, San Diego, CA, USA) specific to the surface markers were used: anti-CD5-Pacific Blue (clone UCHT2) for T cells, anti-CD4-FITC (clone SK3) for CD4+ T cells, anti-CD44-APC/Cy7 (clone IM7) for memory T cells, anti-CD62L-PerCP/Cy5.5 (clone DREG-56) for naive T cells, and CD25-AlexaFluor 700 (clone BC96) as an activation marker.

#### Sample collection and analysis of immune cells

The patients were treated according to the standardized recommendations of the ICU based on Surviving Sepsis Campaign guidelines [16]. Within 24 h after diagnosis of severe sepsis, 8 mL of blood sample was collected. The peripheral blood mononuclear cells (PBMCs) and serum were separated as previously described [15], and dissociated PBMCs (1 × 10^6^) were stimulated overnight with Human T-Activator CD3/CD28 Dynabeads for T-cell expansion and activation (Life Technologies, Grand Island, NY, USA), followed by flow cytometric analysis using a Fortessa Flow Cytometer (BD Biosciences, Franklin Lakes, NJ, USA) to quantify T-cell activation using FlowJo software (FlowJo, Ashland, OR, USA). The white blood cell count was determined from another 2 mL of blood sample collected at the same time. Cells were fractionated for neutrophils and monocytes using a XE-2100 automated hematology analyzer (Sysmex, Kobe, Japan) in the central laboratory of the hospital.

#### CFSE labeling and proliferation analysis

PBMC cells (5 × 10^5^) were labeled with 1 μM CellTrace carboxyfluorescein succinimidyl ester (CFSE) from a cell proliferation kit (Life Technologies) in PBS for 10 minutes at 37˚C. The labeling reaction was quenched by addition of cold Roswell Park Memorial Institute (RPMI) 1640 medium with 10% FCS, and cells were washed twice with PBS with 2% FCS to remove excess CFSE. The CFSE-labeled cells were incubated for 48 h with 2.5 μl of Human T-Activator CD3/CD28 Dynabeads for T-cell expansion and activation (Life Technologies). Cells were harvested and stained with anti-CD4 antibodies for fluorescence activated cell sorter (FACS) analysis based on gating on individual CFSE generations, and the proliferation of CD4+ T cells was analyzed.

#### Cytokine analysis

IL-6 levels in the serum and IL-2 levels in conditioned medium were quantified using a BD FACS array and Inflammation Kit (BD Biosciences, Franklin Lakes, NJ, USA) according to the manufacturer’s recommendations, as previously described [17]. The lower limits of detection were 1.6 pg/mL for IL-6 and 11.2 pg/mL for IL-2.

#### Secondary infection analysis

We performed bacterial and fungal cultivation testing for the patients every 4 to 6 days after ICU hospitalization to address patients' susceptibility to the secondary infections after sepsis. Source and types of bacteria and fungus were categorized and compared between adult and elderly patients.

### Mouse study

#### Mice

Young (6 to 8 weeks) and aged (20 to 22 months) C57/B6 mice were purchased from CLEA, Tokyo, Japan. The mice were housed in groups of five for at least 1 week prior to use. All animal studies were approved by the Tokai University Animal Studies Committee (reference number: 123026).

#### Antibodies

The following murine antibodies (Bio-Legend) specific to the surface markers were used: anti-CD3-FITC (clone 17A2) and anti CD90.2-AlexaFluor 700(clone 30-H12) for T cells, anti-CD4-Pacific Blue and APC (clone GK1.5) for CD4+ T cells, anti-F4/80-PE/Cy7 (clone BN8) and anti-CD11b-APC (clone M1/70) for macrophages, anti-Ly-6G/Ly-6C (Gr-1)-PE (RB6-8C5) for neutrophils, cytotoxic T-lymphocyte antigen 4 (CTLA-4)-APC (clone UC10-4B9) and programmed death 1(PD-1)-APC (clone 29 F.1A12) as negative co-stimulatory molecules, anti-CD44-APC/Cy7 (clone IM7) for memory T cells, anti-CD62L-PE (clone MEL-14) for naive T cells, anti-IL-2-PE (clone JES6-5H4) for IL-2, and CD25-FITC (clone BC69) as an activation marker for T cells. Anti-APC Microbeads were used for CD4+ cell purification (Miltenyi Biotec, Auburn, CA, USA).

#### CLP sepsis model

The CLP model developed by Chaudry *et al*. [18] was used to induce intra-abdominal peritonitis. In brief, the mice were anesthetized with isoflurane and a midline abdominal incision was made. The cecum was mobilized, half-ligated below the ileocecal valve, and punctured once using a 27-gauge needle. The abdomen was closed in two layers, and the mice were subcutaneously injected with 1.0 mL of 0.9% saline containing 2 μg of buprenorphine for analgesia. No fluids or antibiotics were administered to mice that underwent the operation. The mice used for analysis of absolute cell counts and cytokine production were sacrificed at 24 h post surgery. For the survival studies, the mice underwent CLP as described above, and the analysis was discontinued at 7 days after surgery. One-drop blood collection from the cheek plexus of other groups of CLP mice was used for cytokine assays at 3, 6, 12, 18, 24, and 48 h after CLP.

#### Cytokine analysis

Serum cytokine levels were quantified using the BD FACS array as described for the patient study. The lower limit of detection was 1.4 pg/mL for IL-6, 9.6 pg/mL for IL-10, and 2.7 pg/mL for monocyte chemoattractant protein-1 (MCP-1).

#### Harvesting of blood, spleen, and lymph nodes

At approximately 24 h post surgery, blood was collected from the heart of anesthetized mice using a heparinized syringe. PBMCs were separated using Histopaque®-1083 (Sigma-aldrich, St Louis, MO, USA). Next, mice were gently sacrificed by cervical dislocation under sedation, and the spleen and mesenteric lymph nodes were surgically removed and prepared by gently pressing the organs through a 70-μm filter and the cells were then washed and the red cells were lysed. PBMCs and dissociated splenocytes (1 × 10^6^) were stained for cell surface markers and quantified by flow cytometry. CD4+ T cells from splenocytes were purified using a magnetic activated cell sorter (auto-MACS, Miltenyi Biotec) and FACS (Aria II, BD Biosciences).

#### Assay for *ex vivo* activation of T cells

Spleens from young and aged mice were harvested, splenocyte suspensions were prepared, and the cells were stimulated as previously described [19]. The dissociated splenocytes and sorted CD4+ T cells (1 × 10^6^) were stimulated overnight using an anti-CD3 antibody followed by staining to identify CD4+ T cells and quantification of the activation marker CD25 and IL-2 by flow cytometry.

#### CFSE labeling and proliferation analysis

Splenocytes (5 × 10^5^) were labeled with the Cell Trace CFSE Cell Proliferation kit as described for the human PBMC CFSE assay. CFSE-labeled cells were incubated for 48 h with anti-CD3 antibody and CD4+ T cell proliferation was analyzed.

#### Statistical methods

Data were analyzed using IBM SPSS Statistics version 20 (SPSS, Chicago, IL, USA). Two-way analysis of variance (ANOVA) was performed to determine the main effects of sepsis (HDs versus sepsis or sham versus CLP) and aging (adult versus elderly or young versus aged), as well as the interaction between these two factors.

Survival curves were created with the Kaplan-Meier method and compared using the log-rank test. Cox proportional hazards regression analysis was used to determine the net effect of each predictor while controlling for the effects of the other factors by multivariate analyses. The hazard ratio (HR) with 95% CI was used to assess the independent contributions of significant factors. To evaluate the impact of comorbidities on severe sepsis, we also performed sensitivity analysis by excluding patients with comorbidities that were more prevalent in elderly patients than in adult patients; then, we compared the outcomes between the remaining adult and elderly patients with sepsis.

A mixed-effect regression model was used to test the difference in cytokine levels pooled over time in the young and elderly groups (time-adjusted difference). The following covariance structures were considered: unstructured, compound symmetric, first-order autoregressive, and Toeplitz. The covariance structure that provided the best fit according to Akaike’s information criterion was used in the analysis. *P* <0.05 was defined as statistically significant. Results are presented as mean ± SD values.

## Results

### Decreased survival and persistent inflammation in severe sepsis in elderly patients and in aged septic mice

**Figure 1:**
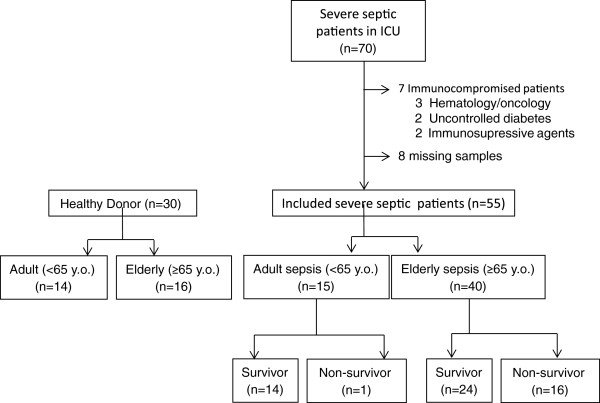
Flow chart of patients included in this study.

**Figure 2:**
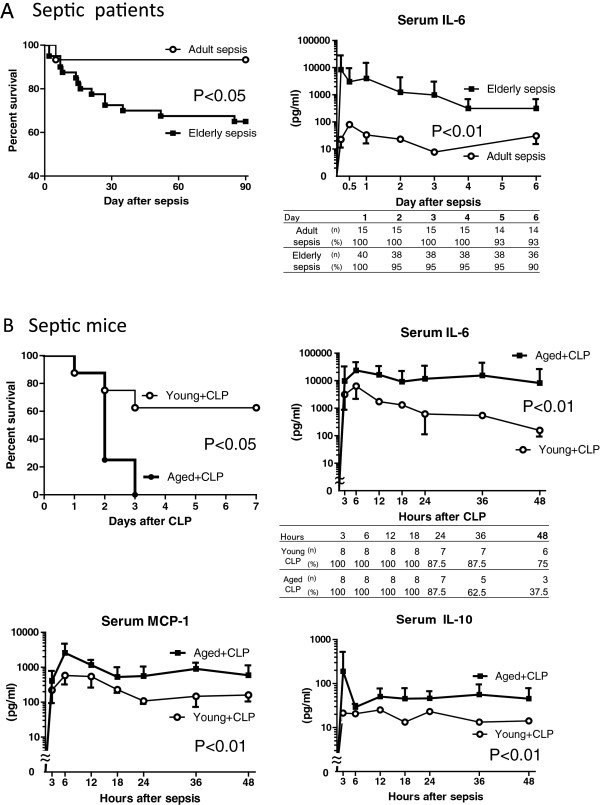
**Decreased survival and prolonged elevation of serum pro- and anti-inflammatory cytokines in severe sepsis in elderly patients. (A)** Left, survival rates of adult and elderly patients with sepsis; right, serum concentrations of IL-6 after diagnosis. **(B)** Upper left, survival rates of aged and young mice after cecal ligation and puncture (CLP) (*P* = 0.015, n = 8 per group); serum concentrations of IL-6 (upper right), monocyte chemoattractant protein-1 (MCP-1) (lower left) and IL-10 (lower right) in young and aged mice after CLP (*P* <0.01).

Fifty-five patients with severe sepsis and thirty HDs were enrolled in the current study (Figure 1). The study participants were divided into two groups based on age: adult (18 to 64 years) and elderly (≥65 years). The clinical characteristics of the respective groups are summarized in Table 1. The 3-month survival of elderly septic patients (60%) was significantly lower than that of adult sepsis patients (93%, *P* <0.05; Figure 2A). Multivariate analysis using Cox proportional hazard regression showed that age was the strongest predictor of 3-month mortality in the patients (HR, 1.07; 95% CI, 1.01, 1.13; *P* = 0.02) (Table 2). The serum IL-6 concentration was consistently higher in elderly sepsis patients than in adult sepsis patients; the mean difference between the groups was 354 to 13,095 pg/mL (*P* <0.01; Figure 2A). We analyzed serum albumin levels at day 1 and day 30 after sepsis in the patients to address the impaired catabolism in the elderly patients. We found decreased serum albumin levels in the septic elderly patients compared to adult patients at both 1 and 30 days after sepsis (*P* <0.05 in elderly survivors, *P* <0.01 in elderly non survivors) (Additional file 1).

**Table 1:** Characteristics of healthy donors and severe septic patients for cytokine analysis in serum

	**Adult patients**	**Elderly patients**	***P*-value**
**Healthy donors, number**	14	16	
Age, y, mean ± SD	24.0 ± 8.1	78.4 ± 8.2	<0.001
Male, n (%)	11 (79)	11 (69)	0.45
Comorbidity, n (%)	0 (0)	5 (31)	0.30
**Severe sepsis, number of patients**	15	40	
Age, y, mean ± SD	34.8 ± 11.9	77.6 ± 7.1	<0.001
Male, n (%)	12 (80)	28 (70)	0.45
Septic shock, n (%)	5 (33)	18 (45)	0.43
Ventilator rate, n (%)	11 (73)	31 (78)	0.75
APACHE II score, mean ± SD	19.2 ± 6.9	22.5 ± 6.9	0.14
APACHE II-age score, mean ± SD	19.0 ± 5.0	15.8 ± 7.6	0.08
SAPS II score, mean ± SD	46.6 ± 17.5	58.6 ± 16.1	0.10
SAPS II-age score, mean ± SD	43.3 ± 9.6	42.7 ± 11.6	0.17
SOFA score, mean ± SD	7.4 ± 4.6	7.3 ± 4.1	0.87
Comorbidity, n (%)	4 (27)	31 (78)	0.01
Types of bacteria detected in first 72 h, n (%)			
Gram-positive bacteria	8 (53)	22 (55)	0.91
Gram-negative bacteria	15 (100)	40 (100)	1.00
Fungi	6 (40)	17 (43)	0.87
Treatment, n (%)			
Adjunctive hydrocortisone, n (%)	1 (7)	2 (5)	0.81
Organ support types			
Ventilator, n (%)	12 (80)	35 (86)	0.48
Hemofiltration, n (%)	3 (20)	10 (25)	0.70
Organ support duration			
Ventilator, d, mean ± SD	11.6 ± 12.3	9.3 ± 16.1	0.68
Hemofiltration, d, mean ± SD	7.3 ± 6.6	9.9 ± 12.8	0.26
ICU stay, d, mean ± SD	21.9 ± 22.6	16.6 ± 16.1	0.57
ICU-free days in 28 days, d, mean ± SD	11.6 ± 9.6	10.9 ± 11.6	0.64
Hospital stay, d, mean ± SD	36.3 ± 35.9	39.6 ± 41.9	0.41

APACHE, Acute Physiology and Chronic Health Evaluation; SOFA, The Sequential Organ Failure Assessment score; SAPS, Simplified Acute Physiology Score.

**Table 2:** Multivariate analysis of clinical features and survival of patients with severe sepsis

**Variable**	***P*-value**	**Hazard ratio**	**95% CI**	
			**Lower**	**Upper**
Age	0.02	1.07	1.01	1.13
SOFA	0.32	1.08	0.93	1.26
Comorbidity	0.99	1.16	0.28	1.26

SOFA, sequential organ failure assessment.

In the murine study, the 7-day survival of the aged mice (0%) was significantly lower than that of the young mice after CLP (63%, *P* <0.05). The majority of the aged CLP-mice rapidly deteriorated at 24 to 48 h after surgery, and all the mice died within 72 h (Figure 2B). The serum IL-6 concentrations were consistently higher in the aged sepsis group than in the young sepsis group; the mean difference between the groups was 11,514 to 28,870 pg/mL (*P* <0.01; Figure 2B). Similar tendencies with regard to kinetics and quantities were observed in analyses of serum MCP-1 and IL-10 levels in the sepsis groups (*P* <0.01, Figure 2B). Persistent inflammation with greatly enhanced myeloperoxidase activity of activated phagocytes was observed in the aged mice from 6 h to 12 h after CLP on *in vivo* imaging, whereas mild inflammation was observed in the young mice at 6 h but not 12 h after CLP (Additional file 2).

### Increased population of neutrophils and macrophages in peripheral blood and lymph nodes of aged mice

**Figure 3:**
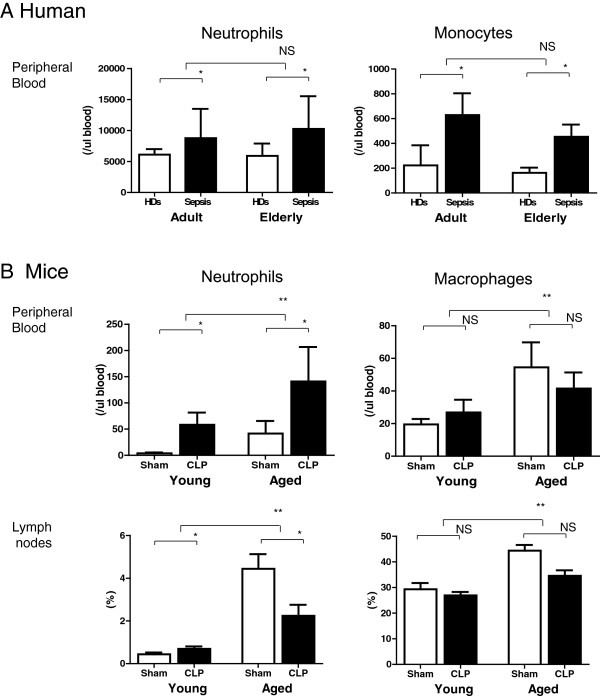
**Increased population of neutrophils and macrophages in peripheral blood and lymph nodes of aged mice. (A)** Neutrophils and monocytes in the peripheral blood of adult and elderly cohorts were counted by an automated hematology analyzer; n = 14 to 40 per group, *P <0.05. **(B)** Populations of neutrophils (Gr1+/CD11b+) and monocytes (F4/80+) in peripheral blood and mesenteric lymph nodes from CLP- and sham-treated mice were identified by flow cytometric analysis; n = 10 to 12 per group, **P <0.01, *P <0.05.

Sepsis induced a significant increase in circulating neutrophils and monocytes in both adult and elderly patients within 24 h after diagnosis, without a significant difference between adult and elderly patients (*P* <0.01; Figure 3A). In contrast to the human cohort, aged mice demonstrated an increased number of neutrophils and macrophages in both the peripheral blood and lymph nodes relative to young mice (Figure 3B).

### Decreased proportion of naïve T cells and increased proportion of memory T cells in severe sepsis in elderly humans and aged mice

**Figure 4:**
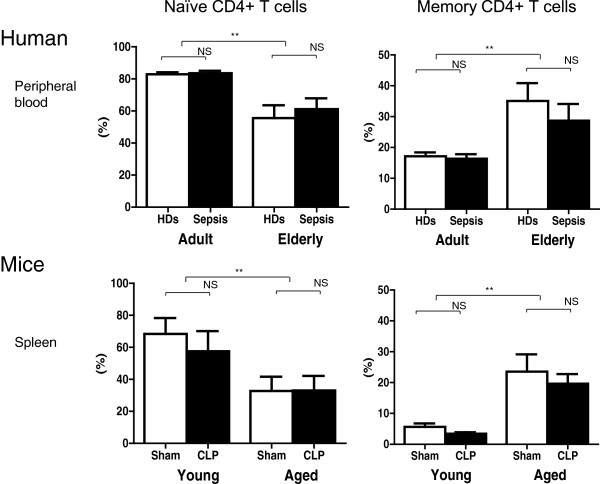
**Decreased proportion of naïve T cells and increased proportion of memory T cells in severe sepsis in elderly patients and aged mice.** Decreased numbers of naïve T cells (CD62L + CD44-) and increased numbers of memory T cells (CD62L- CD44+) among CD4+ T cells were observed in peripheral blood from elderly patients and spleens from aged mice relative to adult humans and young mice by flow cytometric analysis (*P* <0.01); n = 14 to 25 in patients/healthy donors (HDs) and 6 to 8 in mice, per group **P <0.01.

Flow cytometric analysis demonstrated a decreased proportion of naïve T cells (CD62L + CD44-) in peripheral blood from elderly humans and spleens from aged mice relative to adult humans and young mice (Figure 4, *P* <0.01). An increased proportion of memory T cells (CD62L- CD44+) was observed in peripheral blood from elderly humans and spleens from aged mice relative to adult humans and young mice (Figure 4, *P* <0.01).

### Increased expression of negative co-stimulatory molecules in CD4+ T cells in aged mice

**Figure 5:**
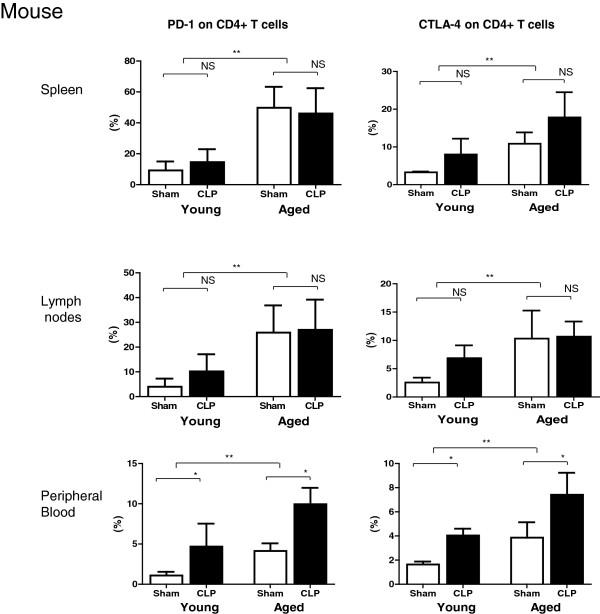
**Increased expression of negative co-stimulatory molecules in CD4+ T cells in aged mice.** The proportions of CD4+ T cells expressing PD-1 (left) and CTLA-4 (right) in the spleen, lymph nodes, and peripheral blood were compared between young and aged cecal ligation and puncture (CLP)- or sham-treated mice at 24 h post surgery; n = 6 per group, **P <0.01, *P <0.05.

Flow cytometric analysis revealed a significant increase in the number of CD4+ T cells expressing PD-1, which is a known negative co-stimulatory molecule, in aged mice relative to young mice (5.7-fold in the spleen and 7.5-fold in the lymph nodes; *P* <0.01; Figure 5). The number of CD4+ T cells expressing CTLA-4, which is also a negative co-stimulatory molecule, was significantly higher in aged mice than in young mice (3.3-fold in the spleen and 4.0-fold in the lymph nodes; *P* <0.01). In peripheral blood, sepsis induced a significant increase of PD-1 and CTLA-4 expression in CD4+ T cells in young and aged mice. The increased number of cells expressing negative co-stimulatory molecules was likely associated with the impaired activation of T cells in aged mice. These age-related immunological changes may contribute to immunosuppression in aged sepsis.

### Impaired activation of T cells in severe sepsis in elderly patients and in aged septic mice

**Figure 6:**
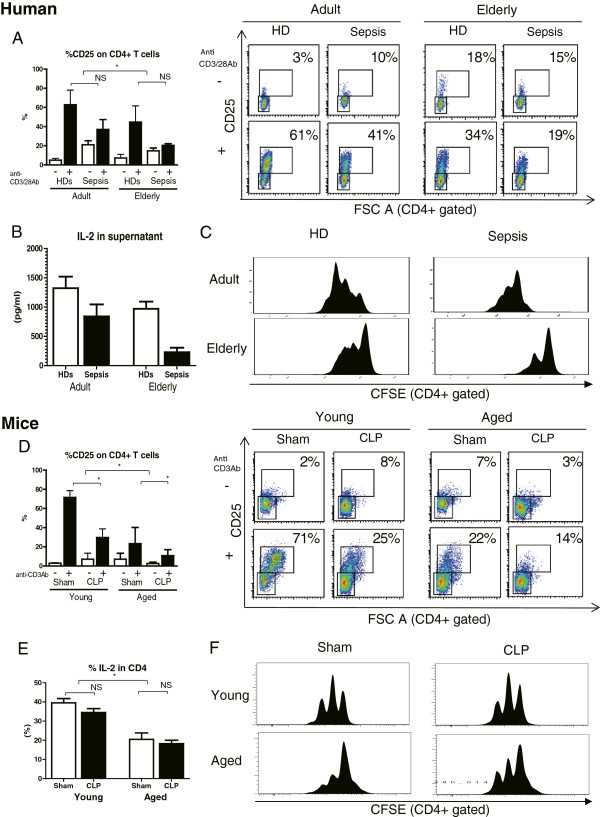
**Impaired *ex vivo *activation, IL-2 production, and proliferation of CD4 + T cells from elderly patients and aged mice with severe sepsis. (A)** Dissociated peripheral blood mononuclear cells were stimulated *ex vivo* overnight with an anti-CD3/CD28 antibody, followed by flow cytometric analysis to quantify T cell activation. **(B)** IL-2 concentration in supernatants from peripheral blood mononuclear cells (PBMCs) with overnight stimulation by anti-CD3/CD28 antibody. **(C)** Profiles of carboxyfluorescein succinimidyl ester (CFSE) fluorescence intensity of activated PBMC cultures. Population doubling of CD4+ T cells can be clearly assessed by the decrease in CFSE fluorescence. Impaired proliferation was observed in CD4+ T cells from elderly healthy donors (HDs) and septic patients; n = 6 to 14 per group, *P <0.05, **P <0.01. **(D)** Dissociated splenocytes were stimulated overnight using the anti-CD3 antibody followed by staining for CD4+ T cells and quantification of the activation marker CD25 by flow cytometry *ex vivo*; n = 6 per group, *P <0.05, **P <0.01. **(E)** Percentage of IL-2-secreting CD4+ T cells on overnight incubation with an anti-CD3 antibody. **(F)** Population doubling of CD4+ T cells from young and aged splenocytes can be clearly assessed by the decrease in CFSE fluorescence. Impaired proliferation was observed in CD4+ T cells from aged sham and CLP mice; n = 6 to 8 per group, *P <0.05.

The patients’ blood samples were collected within 24 h after diagnosis of severe sepsis. The characteristics of the *ex vivo* stimulation assay described below are listed in Additional file 3. Upon activation using an anti-CD3/28 antibody, CD25 expression in CD4+ T cells was lower in adult and elderly patients than in HDs, which suggests that immunosuppression occurred even in the relatively early phase of sepsis. Importantly, the impact of aging on anti-CD3/28-mediated CD25 expression was evident in both HDs and in sepsis patients, which suggests that T-cell activation is impaired in aged individuals irrespective of infection status (Figure 6A). Similar to the results observed in human cells, both aging and sepsis induced a reduction of CD25 expression in mouse CD4+ T cells upon *ex vivo* stimulation by an anti-CD3 antibody (*P* <0.05; Figure 6D). Purified CD4+ T cells of aged mice also showed insufficient activation (Additional file 4).

### Decreased IL-2 production in severe sepsis in elderly patients and in aged mice

We measured the IL-2 concentration in the supernatants of human PBMCs incubated overnight with an anti-CD3/CD28 antibody. In both elderly and septic subjects, IL-2 concentrations were significantly reduced (*P* <0.05) (Figure 6B). We also examined IL-2 expression in CD3/28-stimulated CD4+ T cells of spleens from young and aged mice by flow cytometry. Aged CD4 T cells produced lower amounts of IL-2 (Figure 6E).

### Impaired proliferation of CD4 + T cells in severe sepsis in elderly patients and in aged mice

Population doubling of CD4+ T cells was observed in adult HDs and septic patients as a decrease in CFSE fluorescence. Impaired proliferation was observed in CD4+ T cells from elderly HDs and septic patients (Figure 6C). Similar to the results obtained in elderly humans, impaired proliferation was observed in CD4+ T cells from aged sham-treated mice and aged CLP mice, suggesting age-related T-cell exhaustion (Figure 6F).

### Increased secondary infection in elderly septic patients

To address how the observed T cell exhaustion affects secondary infections after sepsis, we performed bacterial and fungal cultivation testing for the patients after ICU hospitalization. Elderly patients were more susceptible of secondary infection than adult patients, with increased detection of gram-negative bacteria at days 14 to 16 and 28 to 32 after sepsis (*P* <0.05) (Table 3).

**Table 3:** Source and types of secondary infection in the patients after sepsis

	**Day 14 to 16**			**Day 28 to 32**		
	**Adult (n = 14)**	**Elderly (n = 34)**	***P*-value**	**Adult (n = 14)**	**Elderly (n = 29)**	***P*-value**
Source, n (%)						
Sputum	1 (7)	13 (38)		1 (7)	10 (34)	
Blood	0 (0)	4 (12)		0 (0)	1 (3)	
Urine	1 (7)	7 (21)		1 (7)	4 (14)	
Wound	1 (7)	4 (12)		1 (7)	1 (3)	
Total	3 (21)	28 (82)	<0.01	3 (21)	16 (55)	<0.05
Gram-positive, n (%)						
*S. aureus*	0 (0)	1 (3)		1 (7)	1 (3)	
*S. epidermidis*	1 (7)	1 (3)		0 (0)	1 (3)	
MRSA	1 (7)	3 (9)		1 (7)	4 (14)	
Others	4 (29)	6 (18)		0(0)	6 (21)	
Total	6 (43)	11 (32)	0.49	2(14)	12 (41)	0.08
*E. Coli*	0 (0)	3 (9)		0 (0)	0 (0)	
*P. aeruginosa*	0 (0)	2 (6)		0 (0)	1 (3)	
*Klebsiella pneumoniae*	1 (7)	2 (6)		0 (0)	1 (3)	
Acinetobactor	0 (0)	2 (6)		0 (0)	1 (3)	
*S. maltophilia*	0 (0)	5 (15)		0 (0)	5 (17)	
Others	1 (7)	3 (9)		0 (0)	4 (14)	
Total	2 (14)	17 (50)	<0.05	0 (0)	12 (41)	<0.01
*C. albicans*	1 (7)	10 (29)		1 (7)	5 (17)	
*Aspergius fumigatus*	0 (0)	1 (3)		0 (0)	0 (0)	
Total	1 (7)	11 (32)	0.06	1 (7)	5 (17)	0.67

MRSA, methicillin-resistant Staphylococcus aureus; *S. aureus*, Staphylococcus aureus; *S. epidermidis*, Staphylococcus epidermidis; *E. Coli*, Escherichia coli; *P. aeruginosa*, Pseudomonas aeruginosa; *S. maltophilia*, Stenotrophomonas maltophilia; *C. albicans*, Candida albicans.

### Cerebrovascular disease does not affect primary outcomes

To address how comorbidities affected the primary outcome in this study, we performed sensitivity analysis, in which we excluded 10 patients with cerebrovascular diseases because this comorbidity is significantly increased in the elderly. Three-month survival was significantly decreased in elderly patients compared with adult patients (93% versus 60%; *P* <0.05), which is consistent with the primary outcome (Additional file 5). After this exclusion, serum IL-6 levels at 24 h after sepsis and the percentage of CD44+ memory CD4+ T cells were still significantly increased, whereas the percentage of CD62L + naïve CD4+ T cells was significantly decreased in elderly patients with sepsis compared with adult patients with sepsis (*P* <0.05). In the *ex vivo* stimulation study, the percentage of CD25+ activated CD4+ T cells and the IL-2 concentration in the supernatants also significantly decreased in elderly patients with sepsis compared with adult patients with sepsis (*P* <0.05).

## Discussion

We observed persistent inflammation and T-cell exhaustion in aged mice and elderly patients after sepsis with lower survival rates compared to their younger counterparts. It was previously reported that sepsis patients show a systemic inflammatory response, often termed the cytokine storm, in the early stage of sepsis and often develop immunosuppression in the late stage [20-22]. However, our results indicate that excessive inflammation continues for at least 6 days in septic elderly patients and 48 h in aged septic mice, as prolonged elevation of IL-6 in elderly patients and of IL-6, MCP-1, and IL-10 in aged CLP mice was observed (Figure 2A and B, respectively). Therefore, inflammation does not cease in the early stage of sepsis. However, impaired T cell activation, IL-2 production, and proliferation were observed in elderly sepsis patients and aged mice at relatively early stages (24 h after diagnosis in humans and after CLP treatment in mice). It is suggested that prolonged inflammation and T-cell exhaustion occurred simultaneously [11].Although aged mice contained more neutrophils and macrophages in their peripheral blood and lymph nodes than young mice (Figure 3B), there was no significant difference in neutrophil and monocyte number between adult and elderly humans (Figure 3A). Furthermore, there was no age difference in the cytokine production (IL-6, MCP-1, TNF-a, and IL-10) of human PBMCs and mouse macrophages and neutrophils that were stimulated by lipopolysaccharide (LPS) (data not shown). In addition, phagocytosis by neutrophils and macrophages was not different between young and aged mice (data not shown). Although it is possible that an increase in neutrophils or macrophages contributes to enhanced inflammation in aged CLP mice, such an increase does not appear to play a role in the observed effects in humans.

A commonly observed feature of human and mouse sepsis is T-cell exhaustion (Figures 5 and 6). The term exhaustion has been used to describe the state of functional unresponsiveness, replicative senescence, and ultimate physical deletion of T cells during chronic infection in mice and humans [23,24]. We demonstrated that CD4+ T cells in elderly humans and aged mice showed impaired IL-2 receptor (IL-2R) expression (Figure 6A and D), IL-2 production (Figure 6B and E), and cell division (Figure 6C and F). Furthermore, the fraction of PD-1/cytotoxic T-lymphocyte antigen 4 (CTALA-4) expressing-CD4+ T cells was higher in aged mice than in young mice (Figure 5), as we have previously reported in humans [15]. Several features of T-cell exhaustion described above are consistent with previous reports [9,25-29]. T-cell exhaustion is a newly recognized pathophysiologic mechanism of immunosuppression in sepsis that results in failure to activate macrophages and to eradicate invading pathogens, thereby increasing susceptibility to secondary infections, leading to prolonged inflammation. In this study, we also showed that elderly septic patients suffered from secondary infection at 2 and 4 weeks after sepsis, especially infection by gram-negative bacteria (Table 3). Taken together, these findings suggest that T-cell exhaustion in elderly patients may be associated with prolonged inflammation and presumably decreased survival after sepsis.

Although our study demonstrated several similarities in the immune response between patients and mice, some findings in aged mice did not match those in elderly patients. First, CD28 expression in CD4+ T cells was decreased in elderly humans [15], whereas there was no difference between young and aged mice (data not shown). Second, IFN-γ production in CD4+ T cells was decreased in elderly septic patients but increased in aged mice (data not shown). Consistent with our findings, some reports have shown that aging increases IFN-γ production [30-34]. In contrast, other reports have shown that aging reduces IFN-γ production in mice [35-37], whereas Kovacs *et al*. reported that IFN-γ production did not differ between young and aged mice with both sham and experimental burn injury [38]. Therefore, the impact of aging on IFN-γ production is still controversial. Recently, Seok *et al*. reported that genomic responses against endotoxemia, especially in T cells, are quite different between humans and mice [39]. Therefore, species differences in the immune response should be carefully considered when designing translational studies in sepsis.

Several limitations of this study must be noted. The overall sample size of this prospective study was relatively small, and all of the study participants were from a single center. The treatment after sepsis in mice in this study was different from that in patients because no fluids or antibiotics were administered to mice that underwent the CLP operation. In addition, the sample size of the *ex vivo* stimulation study was small.

Another point to discuss is the timing of death after CLP in aged mice. Aged mice subjected to CLP started to die at 24 h after CLP, which raises the possibility that innate immunity was also dysfunctional in these mice. However, some young mice also started to die at this time point (Figure 2B). Klotho knockout mice, in which both innate and adaptive immunity are severely impaired, have been found to die at a much earlier phase at 8 to 12 h after CLP [40]. Therefore, we can assume that the innate immune system was relatively functional in aged mice. The study endpoint of 24 h after CLP may be early to evaluate T-cell immunity; however, 24 h was the latest time point available in this study. Further studies are needed to elucidate these points.

## Conclusion

Elderly septic patients and mice presented prolonged serum IL-6 elevation, impaired CD4+ T cell activation with increased expression of PD-1 and CTLA-4, and impaired IL-2 production and proliferation of CD4+ T cells *ex vivo*. Persistent inflammation and T-cell exhaustion may be associated with decreased survival in aged mice and elderly patients after sepsis.

## Key messages

• Decreased survival and persistent elevation of serum IL-6 levels was observed in severe sepsis in elderly patients and aged septic mice.

• Aging decreased the proportion of naive T cells and increased that of memory CD4+ T cells with high expression of negative co-stimulatory molecules in patients and mice with or without sepsis.

• Aging and sepsis induces T-cell exhaustion with impaired activation, IL-2 production, and proliferation in patients and mice.

• Elderly patients were more susceptible to secondary infection than adult patients, with increased detection of gram-negative bacteria at days 14 to 16 and 28 to 32 after sepsis (*P* <0.05).

• Persistent inflammation and T-cell exhaustion may be associated with decreased survival in aged mice and elderly patients after sepsis.

## Abbreviations

CFSC: carboxyfluorescein succinimidyl ester; CLP: cecal ligation and puncture; CTLA-4: cytotoxic T-lymphocyte antigen 4; FCS: fetal calf serum; HDs: healthy donors; HR: hazard ratio; IFN-γ: interferon-γ; IL: Interleukin; MCP-1: Monocyte chemoattractant protein-1; PBMCs: peripheral blood mononuclear cells; PBS: phosphate-buffered saline; PD-1: programmed death 1; PICS: persistent inflammation immunosuppression, and catabolism syndrome; TNF: tumor necrosis factor.

## Competing interests

The authors declare that they have no competing interests.

## Authors’ contributions

SI and KS collected blood samples from patients and analyzed data. YK and KS performed the flow cytometric analysis. YM measured cytokine levels. KH and SI directed the research schedule. TS organized the research project. All authors read and approved the final manuscript.

## Supplementary Material

Additional file 1 **Decreased serum albumin in elderly non survivors after sepsis.** Serum albumin at day 1 and day 30 after sepsis was measured and analyzed to address impaired catabolism in elderly patients. Decreased serum albumin was observed in the septic elderly patients compared to adult patients at day 1 and 30 days after sepsis; n = 14 to 29 per group, *P <0.05, **P <0.01. Click here for file

Additional file 2 **Extensive and prolonged inflammation in the peritoneal cavity of aged mice after cecal ligation puncture.** Aged mice demonstrated prolonged inflammation, reflected by high luciferase activity, which tracks the myeloperoxidase activity of activated phagocytes and inflammation, at 6 and 12 h after cecal ligation puncture. The mild inflammation observed in the young mice at 6 h after cecal ligation puncture disappeared at 12 h after the procedure. Methods: the IVIS Imaging System (100 Series; Xenogen Corp., Alameda, CA, USA) was used for *in vivo* imaging as previously described [41]. At 0, 6, and 12 h after CLP, mice were anesthetized with isoflurane and intraperitoneally injected with 150 mg/kg luciferin (Biosynth, AG, Switzerland), which traces myeloperoxidase from activated phagocytes. At 10 minutes after luciferin injection, the mice were imaged for 5 minutes. Photons emitted from specific regions were quantified using Living Image software (Xenogen Corporation). The *in vivo* luciferase activity was expressed as photons per second per centimeter square. The color overlay on the image represents the photons per second emitted from the animal in accordance with the pseudo-color scale next to the images. Red represents the highest value in photons per second, whereas blue represents the lowest value. CLP, cecal ligation puncture. Click here for file

Additional file 3 **Characteristics of healthy donors and severe septic patients for *ex vivo* stimulation of T cells.** Click here for file

Additional file 4 **Inactivation of purified CD4+ T cells form aged splenocytes.** The dissociated splenocytes and sorted CD4+ T cells were stimulated overnight using an anti-CD3 antibody followed by staining to identify CD4+ T cells and quantification of the activation marker CD25. Purified CD4+ T cells of aged mice also showed insufficient activation. MACS, magnetic activated cell sorter. FACS, fluorescence activated cell sorter. Click here for file

Additional file 5 Sensitivity analysis by excluding patients with cerebrovascular diseases. Click here for file
