# Supplementary material for: Persistent inflammation and T cell exhaustion in severe sepsis in the elderly
Source: Crit Care. 2014 Jun 24;18(3):R130. doi: 10.1186/cc13941 (PMC4230031; doi:10.1186/cc13941)
Supplement: Additional file 3 — Characteristics of healthy donors and severe septic patients for ex vivo stimulation of T cells. [file cc13941-S3.docx]

|  |  |  |  |  |  |  |  |
| --- | --- | --- | --- | --- | --- | --- | --- |
|  |  | | |  |  |  |  |

| **Additional file 3**  **Characteristics of healthy donors and severe septic patients for *in vitro* stimulation of T cells** | | | | | | | |
| --- | --- | --- | --- | --- | --- | --- | --- |
|  |  |  |  |  |  |  |  |
|  |  |  |  | **Healthy Donors** | | **Septic patients** | |
|  |  |  |  | **Adult**  **(n = 14)** | **Elderly (n = 16)** | **Adult (n = 6)** | **Elderly (n = 12)** |
|  | Age, y (mean ± SD) | | | 24.0 ± 8.1 | 78.4 ± 8.2 | 26.0 ± 9.3 | 80.3 ± 12.1 |
|  | Male, n (%) | | | 11 (79) | 11 (69) | 15 (83) | 9 (75) |
|  | Septic shock, n (%) | | | - | - | 2(33) | 4(33) |
|  | APACHE II score (mean ± SD) | | | - | - | 23.8 ± 6.3 | 21.8 ± 6.6 |
|  | SOFA score (mean ± SD) | | | - | - | 8.2 ± 4.9 | 7.8 ± 5.7 |
|  | Co-morbidity, n (%) | | | 0 (0) | 5 (31) | 0 (0) | 5 (42) |
