# Supplementary material for: Persistent inflammation and T cell exhaustion in severe sepsis in the elderly
Source: Crit Care. 2014 Jun 24;18(3):R130. doi: 10.1186/cc13941 (PMC4230031; doi:10.1186/cc13941)
Supplement: Additional file 5 — Sensitivity analysis by excluding patients with cerebrovascular diseases. [file cc13941-S5.docx]

| **Additional file 5 Sensitivity analysis excluding patients with cerebrovascular diseases** | | | | | | | | | |  |
| --- | --- | --- | --- | --- | --- | --- | --- | --- | --- | --- |
|  |  |  |  |  | |  |  |  |  |  |
|  | | | Primary outcomes | | | |  | Sensitivity analysis excluding patients with cerebrovascular diseases | | |
|  | | | Severe Sepsis | | | p value |  | Severe Sepsis | | p value |
|  | | | <65 y.o. | | ≥65 y.o. |  |  | <65 y.o. | ≥65 y.o. |  |
|  |  |  | (n=15) | | (n=40) |  |  | (n=15) | (n=30) |  |
| Three-month survival, n (%) | | | 14 (93) | | 24 (60) | <0.05 |  | 14 (93) | 20 (60) | <0.05 |
| Serum IL-6 at 24hrs after sepsis (pg/ml) | | | 13429.4 | | 85 | <0.01 |  | 13429.4 | 78.2 | <0.01 |
| % CD62L+ in CD4+ T cells, (%) | | | 83.5 | | 61.2 | <0.01 |  | 83.5 | 58.8 | <0.01 |
| % CD44+ in CD4+ T cells, (%) | | | 16.3 | | 28.7 | <0.05 |  | 16.3 | 29.8 | <0.05 |
|  | | | (n=6) | | (n=12) |  |  | (n=6) | (n=12) |  |
| % CD25+ in CD4+ T cells, (%) | | | 37 | | 20 | <0.05 |  | 37 | 20 | <0.05 |
| IL-2 concentration in supernatants, (pg/ml) | | | 841.6 | | 228.7 | <0.05 |  | 841.6 | 228.7 | <0.05 |
|  | | |  | |  |  |  |  |  |  |
